# Role of lncRNA Has2os in Skeletal Muscle Differentiation and Regeneration

**DOI:** 10.3390/cells11213497

**Published:** 2022-11-04

**Authors:** Wanxin Chen, Weicai Chen, Peng Liu, Shiyu Qian, Shuang Tao, Mengchun Huang, Wanyi Xu, Cuiping Li, Xiaoyan Chen, Huizhu Lin, Zhenshu Qin, Jianxi Lu, Shujuan Xie

**Affiliations:** 1 Biotherapy Center, The Third Affiliated Hospital of Sun Yat-Sen University, Guangzhou 510630, China; 2 Laboratory Medicine, The Third Affiliated Hospital of Sun Yat-Sen University, Guangzhou 510630, China; 3 Department of Public Health and Preventive Medicine, School of Medicine, Jinan University, Guangzhou 510632, China; 4 Department of Trauma Orthopaedics, Chenzhou First People’s Hospital Affiliated to South China University, Chenzhou 423000, China; 5 Vaccine Research Institute of Sun Yat-Sen University, The Third Affiliated Hospital of Sun Yat-Sen University, Guangzhou 510630, China

**Keywords:** Has2os, lncRNA, skeletal muscle differentiation, JNK/MAPK, regeneration

## Abstract

Long non-coding RNAs (lncRNAs) regulate a series of physiological processes and play an important role in development, metabolism and disease. Our previous studies showed that lncRNAs involved in skeletal muscle differentiation. Here, we demonstrated that lncRNA Has2os is highly expressed in skeletal muscle and significantly elevated during skeletal cell differentiation. The knockdown of Has2os inhibited myocyte fusion and impeded the expression of the myogenic factors MyHC and Mef2C. Mechanically, Has2os regulates skeletal muscle differentiation by inhibiting the JNK/MAPK signaling pathway. Furthermore, we also revealed that Has2os is involved in the early stage of regeneration after muscle injury, and the JNK/MAPK signaling pathway is activated at both protein and mRNA levels during early repair. Our results demonstrate the new function of lncRNA Has2os, which plays crucial roles during skeletal muscle differentiation and muscle regeneration, providing a basis for the therapy of lncRNA-related muscle diseases.

## 1. Introduction

Skeletal muscle is the largest metabolic and endocrine organ of human body, accounting for about 40% of the body weight and playing a vital role in human movement, protein storage, metabolism and internal organ protection, and skeletal muscle is vital for body homeostasis [1,2]. A mountain of evidence shows that skeletal muscle differentiation is precisely regulated by multiple signaling pathways and myogenic regulators (MRFs) [3,4], including MyoD, Myf5 and myogenin (MyoG), as well as their co-regulator myocyte enhancer 2 (Mef2) family [5,6]. It is well known that mitogen-activated protein kinase (MAPK) signaling participates in various biological processes and is vital for muscle function. Our previous reports show that MyoD interplay with JNK/MAPK signaling pathway determined myoblasts differentiation [7], and Mef2A-p38/MAPK, together with noncoding RNA miR-101 and lncRNA MALAT1, regulates the process of muscle development [8]. Moreover, the ERK/MAPK signaling pathway also takes part in this process [9,10].

Long non-coding RNAs (lncRNAs) constitute a new class of non-coding RNAs that are longer than 200 nucleotides, have been identified in various cells, and play important roles in normal physiology as well as in many diseases [11,12]. Many studies have reported that lncRNAs can participate in myocyte proliferation and differentiation process [13,14], and they play an important role in skeletal muscle development. For example, H19, one of the earliest known imprinted lncRNA, is strongly repressed after birth in all mouse tissues, but it remains expressed in adult skeletal muscle and heart [15]. lncRNA ZFP36L2-AS is specifically enriched in skeletal muscle and inhibits myoblast proliferation while promoting myoblast differentiation [16]. LncMGPF is highly expressed in muscle and promotes the myogenic differentiation of muscle cells in vivo and in vitro by acting as a molecular sponge of miR-135a-5p [17]. Additionally, several lncRNAs have been reported to shape muscle and functional in muscle homeostasis [18], including the MyoD-related lncRNAs linc-MD1 [19] and LncMyoD [20,21], MyoG-related lncR-Myoparr [22], muscle-specific lncR-Irm [23], lincYY1 [24,25], lncRNA Dum [26], and linc-RAM [27]. However, the identification of functional lncRNAs in the growth and development of skeletal muscle is yet to be fully elucidated. Therefore, the identification and study of lncRNAs that exert key regulatory functions during skeletal muscle differentiation and regeneration are important for refining the epigenetic regulatory network during skeletal muscle development.

Our recent study characterized the expression and epigenetic modification profiles of lncRNAs that display temporal expression in mouse myoblasts and differentiated myotubes [28]. Their several lncRNAs caught our attention, including Has2os, an lncRNA that has not been studied and is harbored in the Hyaluronan synthase 2 (Has2) gene. Intriguingly, Has2 is a skeletal-muscle-specific expressed gene that is reported to play a crucial role in skeletal growth, patterning, chondrocyte maturation and synovial joint formation in the developing limb [29]. Additionally, endogenous hyaluronan synthesis is required for myogenic differentiation, and a hyaluronan-rich microenvironment in the limbal stem cell niche regulates limbal stem cell differentiation [30,31,32]. It is believed that the function of lncRNAs might have some relationship with their neighboring genes by cis-acting mechanisms [33]. This information led us to hypothesize that lncRNA Has2os probably contributes to muscle tissue development.

In this study, we characterized the expression pattern of lncRNA Has2os, and found it to be highly expressed in skeletal muscle and highly conserved in both human and mouse genomes. Our experimental data show that Has2os steadily increases during skeletal muscle differentiation, and the knockdown of its expression impairs this process, suggesting Has2os serves as a differentiating promote factor. Meanwhile, Has2os is also involved in an early state of regeneration after skeletal muscle injury. Furthermore, we found that Has2os participates in these processes by regulating the JNK/MAPK signaling pathway. Our data reveal the new regulatory network of lncRNA Has2os, especially in skeletal muscle, which may help to further understand the mechanism underlying the epigenetic regulation of skeletal muscle development and regeneration.

## 2. Materials and Methods

### 2.1. Cell Culture and Differentiation Induction

The C2C12 mouse myoblast cells were purchased from the Cellular Library of the National Collection of Authenticated Cell Cultures (Shanghai, China). The cells were cultured in growth medium (GM)-Duchenne’s modified Eagle medium (DMEM, Gibco, Carlsbad, CA, USA) with 10% fetal bovine serum (FBS, Gibco), penicillin (100 U/mL), and streptomycin (100 µg/mL) at 37 °C in a humidified chamber supplemented with 5% CO_2_. When C2C12 cells reached about 90% confluency, the GM was replaced with differentiation medium (DM), containing 2% horse serum (HyClone, Logan, UT, USA). The medium was changed every two days. Post-differentiation, myotubes’ morphology was observed using phase-contrast microscopy. Phenotypic differentiation was typically observed during 3 days of culturing the cell in DM, while more significant myotube formation was observed after 5 days. Cells were collected on the indicated time points for analysis.

### 2.2. RNA Extraction and qRT-PCR Assays

Total RNA was extracted from the collected cells with Trizol reagent (Invotrogen) according to the manufacturer’s instructions, and the RNA was measured for its concentration and quality, ensuring that the appropriate concentration of A_260_/A_280_ was between 1.8 and 2.0. Then the cDNA was reverse transcribed with the HiScript III RT SuperMix for qPCR (+ gDNA wiper) (Vazyme, Nanjing, China, R323-01) according to the manufacturer’s instructions. qRT-PCR was performed by Roche LightCycler 480 SYBR qPCR Master Mix (Vazyme). PCR amplification system (total volume: 10μL): cDNA 2μL, SYBR qPCR Master Mix 5μL, primers 1 μL, RNase-Free Water 2 μL. PCR amplification procedure: 1. Pre-denaturation 95 °C for 30 s; 2. Denaturation: 95 °C for 10 s, annealing 60 °C for 30 s, a total of 45 cycles; 3. Draw the melting curve: 95 °C for 15 s, 60 °C for 60 s, 95 °C for 15 s. Relative gene expression levels were measured using 2−ΔΔCt method. A list of the primers is provided in Table 1.

### 2.3. Western Blot

For cultured cells, cells were harvested washed with ice-cold PBS and lysed using RIPA lysis buffer (ShanghaiWanshengHaotian Biological Technology), adding protease inhibitor (Roche, Rotkreuz, Switzerland) and phosphatase inhibitor (Roche). For mice muscle tissues, tissues were lysed in RIPA lysis buffer adding protease inhibitor (Roche) and phosphatase inhibitor (Roche) and grinded using a Homogenizer (Servicebio, Wuhan, China). The lysate was incubated on ice for 20 min and centrifuge at 13,000 rpm for 10 min, and the resulting supernatant was collected as protein samples. Protein concentration was determined by BCA protein quantification kit (KeyGEN), and a proportion of sample buffer was added, and denatured for 10 min at 99 °C. The equivalent total 30 ug of protein extract was separated by SDS-PAGE (10% acrylamide separating gel and 4% acrylamide stacking gel containing 10% SDS), and transferred to nitrocellulose filter membrane. Membranes were blocked with 5% skim milk for 1 h at room temperature and incubated in the corresponding primary antibodies (diluted at 1:2000 with primary antibody dilution (Beyotimee, Shanghai, China)) overnight at 4 °C. The next day, after three times washing, membranes were incubated with secondary antibodies (diluted at 1:5000 with primary antibody dilution) at room temperature for 1 h. Repeating the wash three times, the membranes proceed to image by BioRad multiple-function imager. The blotting results of JNK, p38, ERK, and C-Jun were re-blotted from the membrane previously incubated with p-JNK, p-p38, p-ERK, aknd p-C-Jun after incubation in stripping buffer (Stripping buffer, Beyotime, P0025) according to manufacturer’s instructions. The following antibodies were used in this study: MyHC (MAB4470) was obtained from R&D Systems, Mef2C (#5030S), p-JNK (#4671S), JNK (#9258S), p-p38 (#4511S), p38 (#9212S), p-ERK (#4370S), ERK (#4695S), p-C-Jun (#9261), C-Jun (#9165), MKK4 (#9152), MKK7 (#4172), and GAPDH (#2118) were obtained from Cell Signaling Technology. Antibodies for MEKK1 (ab69533) and MEKK2 (ab40805) were obtained from Abcam. Pax7 antibody was obtained from Proteintech Ca (#20570-1-AP). MyoD (#SC-760), MyoG (#SC-12732), and Has2 (#SC-364239) were obtained from Santa Cruz Biotechnology. Secondary antibodies for Rabbit (#7074s) and Mouse (#7076s) were obtained from Cell Signaling Technology. Gray value of each image was quantified by Image J analyzer. Results were presented as mean ± SEM. An unpaired, two-tailed Student’s *t*-test was used for comparisons between two groups. The statistical significance of difference between two means was calculated with the *t*-test, * *p* < 0.05, ** *p* < 0.01, *** *p* < 0.001, **** *p* < 0.0001.

### 2.4. Gene Knockdown

To knock down Has2os, designed siRNAs targeting Has2os (si-Has2os-#1, si-Has2os-#2, si-Has2os-#3) and control siRNAs were synthesized by Gemma Biological Limited (JiangSu, China). C2C12 cells were cultured in 12-well plates, next day when the cells reached 30–40% confluency transfected with siRNAs using Lipofectamine 2000 (Invitrogen, Waltham, MA, USA) according to manufacturer’s instructions. Transfected cells were maintained in growth medium for two days and shifted into DM for another three days, after which cells were harvested for analysis. All siRNA sequences used in this study are listed in Table 2.

### 2.5. Immunofluorescence

Transfected C2C12 cells were plated into Miliicell EZ slide (Millipore, Burlington, MA, USA, PEZGS0816) and maintained in growth medium for two days, then shifted into DM for another three days. Cells were fixed in 4% PFA for 20 min followed by permeabilization (0.1% Triton X-100 in PBS) for 5 min and blocking in 3% bovine serum albumin (BSA) in PBS for 1 h at room temperature. Then incubated with primary antibody against MyHC (R&D, MAB4470, 4 ug/mL) for 4 °C overnight. After being washed three times with PBS, cells were stained with secondary antibodies (Alexa Fluor^®^ 594, donkey anti-mouse, Invitrogen, A-21203, 1:1000) for 1 h at room temperature. After staining with Hoechst 33342 (Life Science Tech, H1399) for 10 min at room temperature, the coverslip was installed and imaged by a confocal microscope (LSM880).

### 2.6. Lucierase Reporter Assays

To detect the effect of lncRNA Has2os on JNK signaling pathway activity, C2C12 cells were seeded in 96-well plate and transfected with siRNAs (si-NC, si-Has2os-#1, si-Has2os-#2, si-Has2os-#3), together with pathway reporter plasmids (JNK pathway reporter plasmids or control plasmids) using the lipofectamine 2000 reagent (Invitrogen), then cultured for 2 days in growth medium and replaced with differentiation medium for 3 days. Cells were next lysed in passive lysis buffer and luciferase activities were measured with a Dual Luciferase Assay Kit (Promegaa, Madison, WI, USA). Has2os siRNAs and pathway reporters were transfected with the dose of 100 nM/well and 50 ng/well in 96-well plates, respectively. All luciferase assays were repeated at least three times and performed in triplicate each time.

### 2.7. Skeletal Muscle Injury Regeneration Model Was Established

Muscle injury was induced by the injection of cardiotoxin (CTX) in saline intramuscularly as a single dose of 25 uL of 10 μM into the right tibial forelimb of the mice (C57BL/6J mice). Similarly, the same volume of saline was injected into the corresponding left tibial forelimb of the same mice. Five mice were used in this studied. All processes were achieved under anesthesia by the injection of 2% pentobarbital sodium (45 mg/kg BW) intraperitoneally. After 3 days after muscle injury, animals were anesthetized and the muscle specimens were collected. The muscle specimens were subjected to Hematoxylin-eosin staining (HE) and samples were then frozen in liquid nitrogen for RNA and protein extraction. Mice were housed in the animal facility and had free access to water and standard rodent chow. The animal study was reviewed and approved by the Institutional Animal Care and Use Committee of Third Affiliated Hospital of Sun Yat-sen University.

### 2.8. Statistical Analysis and Processing

Experimental data were analyzed by Graphpad Prism (version 9). The charts were made using Adobe PhotoshopCS5 and Adobe Illustrator. Gray value of each image was quantified by Image J analyzer. All the quantitative results are presented as mean ± SEM. Two-tailed Student’s *t*-test was performed for comparison analysis, * *p* < 0.05, ** *p* < 0.01, *** *p* < 0.001, **** *p* < 0.0001.

## 3. Results

### 3.1. lncRNA Has2os Is Highly Expressed in Skeletal Muscle

**Figure 1:**
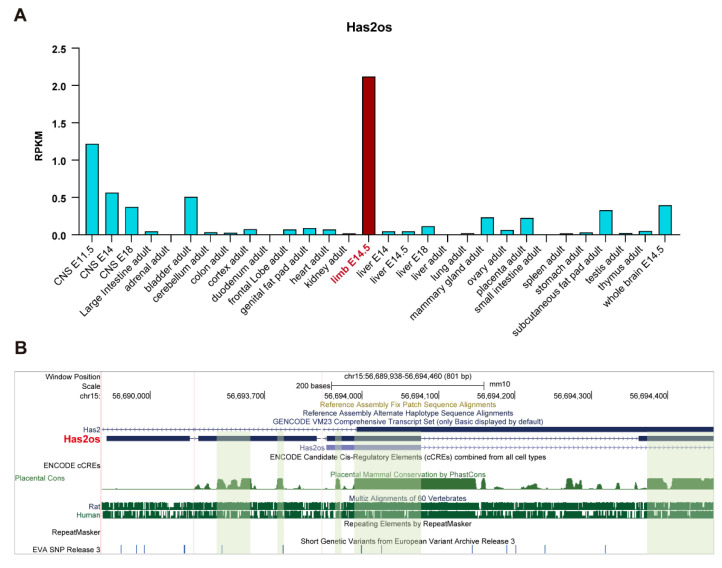
lncRNA Has2os was highly expressed in skeletal muscle. (**A**). lncRNA Has2os was significantly highly expressed in skeletal muscle. Vertical coordinate is RPKM (reads per kilobase of transcript per million reads mapped). Data were obtained from the NCBI database (https://www.ncbi.nlm.nih.gov, Accessed on 12 February 2022). (**B**). lncRNA Has2os is harbored in the Has2 gene, and conserved across multiple vertebrate species especially in human and mice. Data were obtained from UCSC genome browser (http://www.genome.ucsc.edu, Accessed on 15 October 2021).

Our previous study showed that lncRNA Has2os is involved in myogenesis [28], but its function remains to be expounded. Firstly, we sorted its expression profile in a public database-NCBI database (NR_002874). The Mouse ENCODE transcriptome data show that Has2os has an extremely highly expression in muscle limbs but much lower Has2os expression levels in other tissues and organs (Figure 1A). The high expression of Has2os in mouse muscle tissue suggests it is a tissue specifically expressing lncRNA and may participate in the regulation of skeletal muscle homeostasis. Furthermore, we analyzed the location of the Has2os genome and its conservation using a UCSC genome browser. The results revealed that the Has2os gene is located on chromosome 15 and consists of four exons with lengths of 485 nt harbored in Has2, which is a protein coding gene. Additionally, Has2os is conserved across many special genes. As shown in Figure 1B, there are a couple of conserved blocks in the exons of Has2os as measured in the evolutionary conservation of 60 vertebrate species, especially in humans and mice. Meanwhile, we compared its sequence across 60 vertebrate species; the MAF (multiple alignment format) file exhibited multiple alignments at the DNA level between entire genomes and shows that Has2os is highly conserved between mice, rats and humans (Supplementary file S1). It is agreed that more conserved lncRNAs always play more important roles, suggesting that Has2os may play a key role in mammalian muscle.

### 3.2. Expression Pattern of Has2os in Skeletal Muscle Differentiation

**Figure 2:**
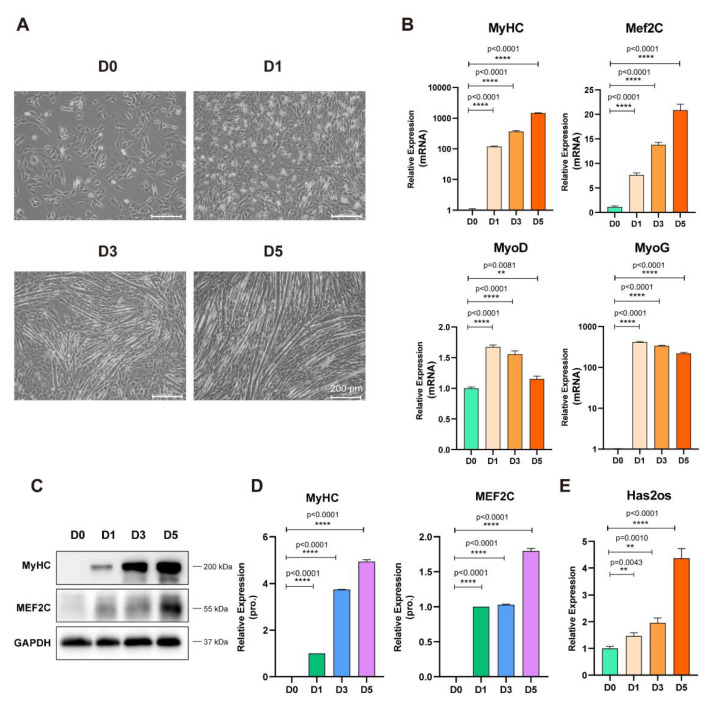
Has2os expression was increased in differentiated muscle cells. (**A**). The morphological changes in C2C12 cells before and after differentiation. D0 represents cells in growth medium, and D1, D3, and D5 represent cells switched into differentiation medium for 1, 3, or 5 days. Scale bar = 200 μm. (**B**). The mRNA expression levels of the myogenic marker MyHC, Mef2C, MyoD, and MyoG were measured before and after the differentiation of C2C12 cells. (**C**). The protein expression levels of myogenic markers MyHC and MEF2C were detected by Western blot. GAPDH was the internal control. (**D**). Relative expression in (**C**) were calculated. (**E**). The expression levels of Has2os in D0, D1, D3, and D5. GAPDH was the internal control. Values were presented as means ± SEM. The statistical significance was calculated by *t*-test.

Based on significantly high Has2os expression in muscle tissue and its high conservation in the mouse and human genomes, we further investigated the expression pattern of Has2os in skeletal muscle differentiation. We utilized C2C12 mouse myoblasts to mimic skeletal muscle differentiation [34]. The differentiation status of C2C12 myoblast cells showed that they proliferated in the presence of serum and differentiated upon serum withdrawal, which were observed through significant morphology changes with gradually fused myotubes. On day 5, there was a pronounced bundle of muscle filament formation (Figure 2A). Total cellular RNAs and protein were collected at corresponding time points (day 0, day 1, day 3, day 5). The RNA expression levels of muscle differentiation markers—MyHC, Mef2C, MyoD, and MyoG—were measured by qRT-PCR. As Figure 2B shows, the RNA expression levels of MyHC and Mef2C showed a continuous increasing trend and a significant increase in abundance (*p* < 0.0001), while the RNA expression levels of MyoD and MyoG showed a trend of first increasing, downregulating from D0 to D5, and then peaking at D1 (*p* < 0.0001, Figure 2B). Meanwhile, the protein levels of MyHC and MEF2C were measured by Western blot. MyHC and MEF2C protein expression levels gradually increased along with differentiation, the relative quantitative analysis of the proteins was highly significant (*p* < 0.0001, Figure 2C,D) and consistent with their mRNA results. These results confirm the success of the model construction of skeletal muscle differentiation induced by mouse myoblasts in vitro. Next, we detected the expression level of Has2os during skeletal muscle differentiation. Compared with in undifferentiated myoblasts, Has2os was gradually upregulated during this process, with a 4.367-fold increase on day 5 (Figure 2E). Moreover, we examined the mRNA and protein expression levels of its host gene Has2 during skeletal muscle differentiation, and found that both Has2 mRNA and its protein were gradually upregulated with differentiation (Supplementary Figure S1), which is consistent with Has2os, as well as previously reported Has2 [30]. Thus, the high expression of Has2os indicated that it might be a potential regulatory factor in the differentiation process of mouse myoblasts.

### 3.3. Knockdown of Has2os Significantly Blocked Muscle Differentiation

**Figure 3:**
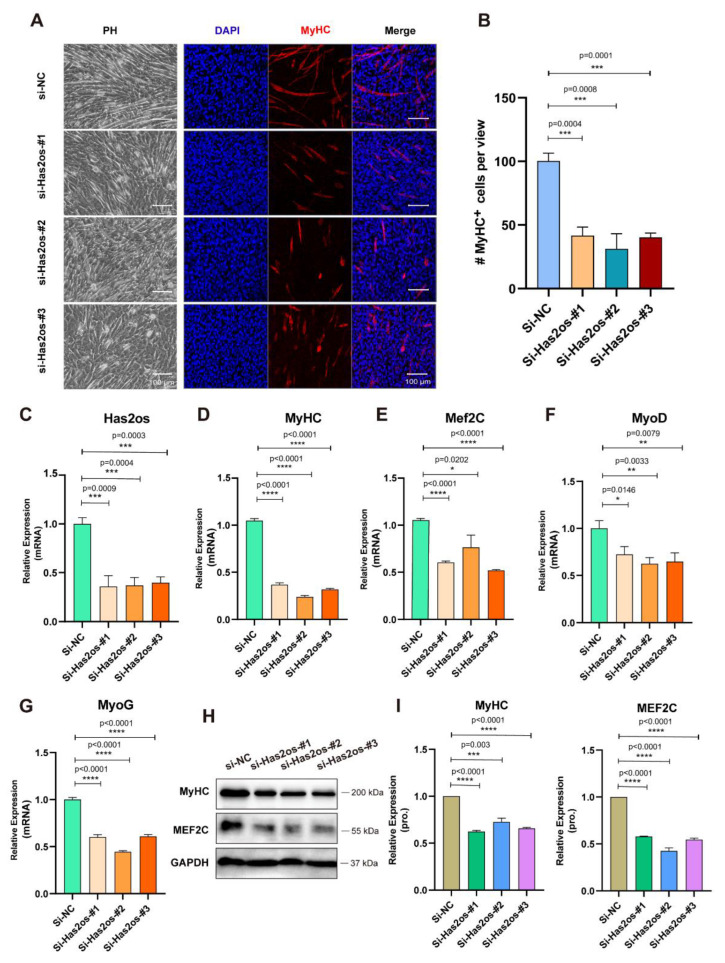
Has2os knockdown inhibited myoblast differentiation. (**A**). Inhibition of myocyte fusion upon Has2os knockdown. Transfected cells were maintained in growth medium for two days and shifted into DM for differentiating another three days, then cells were photographed by microscope to show the morphological changes. Immunofluorescence microscopy analyzed the expression of myogenesis marker MyHC as red fluorescence in C2C12 cells transfected with si-Has2os or si-NC. Scale bar = 100μm. (**B**). MyHC+ cells were calculated based on staining described in panel (**A**). The results are normalized to those of with si-NC. (**C**). The qPCR results showed a successful Has2os knockdown by si-RNA. (**D**–**G**). The qPCR results showed the downregulation of the differentiation marker MyHC, Mef2C, MyoD, and MoyG after Has2os knockdown. (**H**). The Western blot results showed the downregulated myogenesis marker MyHC and MEF2C after Has2os knockdown. (**I**). Relative expression in (**H**) were calculated. GAPDH was the internal control. Values were presented as means ± SEM. The statistical significance was calculated by *t*-test.

To investigate the function of Has2os during skeletal myogenic differentiation, three siRNAs were designed for inhibiting Has2os expression (si-Has2os-#1, si-Has2os-#2, and si-Has2os-#3). C2C12 myoblast cells were effectively transfected with siRNAs in growth medium, proliferated to a high confluence, and then replaced with a differentiation medium for three days. The morphology of Has2os-depleted (si-Has2os-#1, si-Has2os-#2, si-Has2os-#3) cells varies greatly compared to control si-NC cells with less confluent myotubes. Meanwhile, the immunofluorescence microscopy analysis of morphological change and the expression of MyHC show that the number of muscular tubes after si-Has2os was significantly less than si-NC (Figure 3A). MyHC-positive cells were calculated, and statistics showed that the number of MyHC-positive muscular tubes in Has2os-depleted groups was much less than that of the control group (Figure 3B). Additionally, RNA expression levels of Has2os were significantly decreased in si-Has2os cells compared to si-NC cells (Figure 3C), which were measured by qRT-PCR, indicating that Has2os had been successfully knocked down in the myocytes of the three experimental groups. Moreover, after Has2os knockdown, we examined the mRNA and protein expression levels of Has2 and found that the mRNA and protein expression levels of Has2 were not altered (Supplementary Figure S2).

Furthermore, we tested the mRNA expression levels of myogenic markers, MyHC, Mef2C, MyoD, and MyoG, which were all significantly downregulated in the Has2os-depleted groups (Figure 3D–G). Meanwhile, after si-Has2os, MyHC, and MEF2C protein expression levels significantly decreased (Figure 3H). Calculating the gray values of MyHC and MEF2C proteins also showed reduced results (Figure 3I). In addition, we examined mRNA and protein expression levels of Pax7 and ki67, and found that the expression of the muscle-specific proliferative gene Pax7 was promoted after Has2os knockdown, as well as the expression of the cell-proliferation marker molecule ki67 (Supplementary Figure S3). These results indicate that Has2os knockdown inhibited myogenic differentiation, inferring that Has2os can positively regulate the myogenic differentiation process.

### 3.4. Has2os Regulates Skeletal Muscle Differentiation through the JNK/MAPK Signaling Pathway

**Figure 4:**
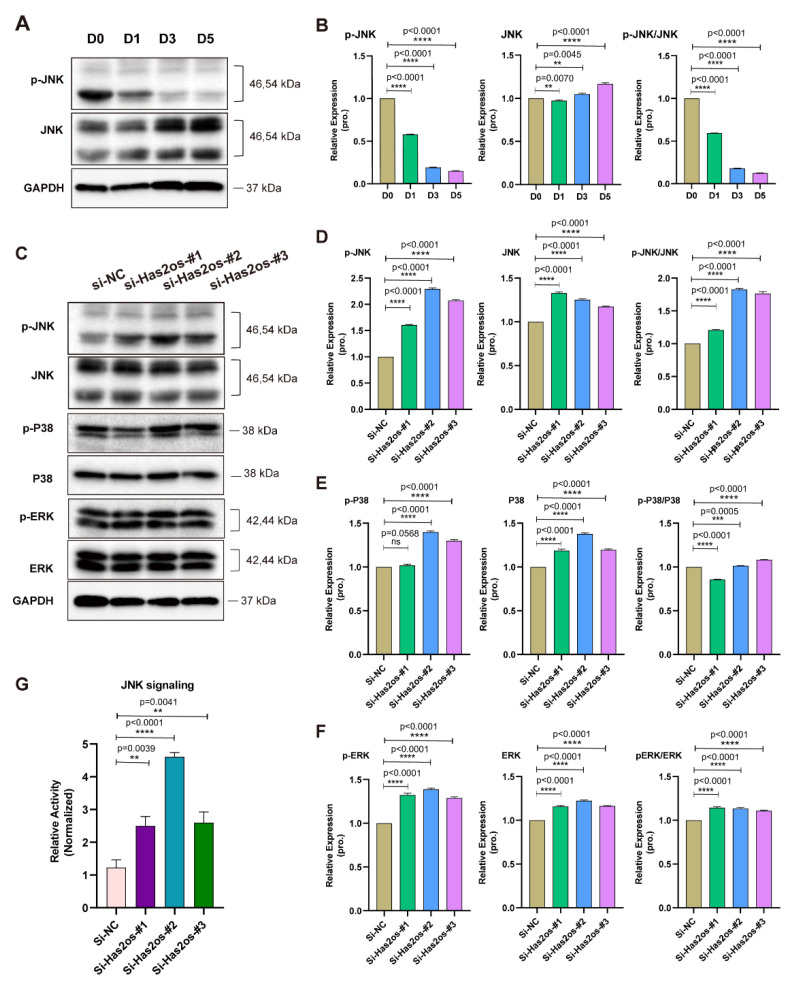
Knockdown of Has2os affected the JNK signaling pathway. (**A**). The JNK signaling pathway is inhibited during skeletal muscle differentiation. The protein expression levels of p-JNK and JNK were detected by Western blot. D0 represents the C2C12 cells in growth medium and D1, D3, and D5 represent cells switched into differentiation medium for 1, 3, or 5 days. (**B**). Relative expression in (**A**) were calculated. (**C**). Western blot analysis shows the JNK signaling pathway was activated upon Has2os knockdown, meanwhile, the p38 and ERK signaling pathways slightly changed. (**D–F**). Relative expression in (**C**) were calculated. GAPDH was the internal control. (**G**). Relative activity of JNK signaling in C2C12 cells that were transfected with si-Has2os or si-NC. The control plasmid was used as the internal control. The results are normalized to those of with si-NC. Values were presented as means ± SEM. The statistical significance was calculated by *t*-test; ns, no statistical significance.

The MAPK signaling pathway is one of the important signaling systems of living organisms, and is reportedly related to cell proliferation, differentiation, and apoptosis. In our previous studies, the MAPK signaling pathway played a crucial role in the proliferation of C2C12 myoblasts. We tested the levels of phosphorylation and total JNK protein expression during C2C12 cell differentiation. As shown in Figure 4A, the levels of the p-JNK protein sharply decrease as cell differentiation progresses, while the total JNK protein increases slightly. By calculating the grayscale values of p-JNK and JNK proteins, it was found that the ratio of the two gradually decreased with differentiation (Figure 4B). To further explore whether Has2os could regulate the MAPK signaling pathway, we detected three important MAPK branching signaling pathways—JNK/MAPK, p38/MAPK, and ERK1/2/MAPK—in the Has2os-depleting and NC cells. As shown in Figure 4C, compared with si-NC cells, the protein expression levels of phosphorylated JNK (p-JNK) were all elevated in si-Has2os cells. The total JNK protein did not change, while another two MAPK branching signaling phosphorylated proteins, p-p38 and p-ERK, as well as their corresponding total proteins, were not clearly different in the si-Has2os-#1-, si-Has2os-#2-, and si-Has2os-#3-transfected cells from the si-NC cells. The relative quantitative analysis of the proteins also showed results consistent with the Western blots (Figure 4D–F). Furthermore, to verify whether Has2os could affect the JNK signaling pathway, we performed a pathway reporter assay. Interestingly, compared with the negative group (si-NC), knocked down Has2os by all the three siRNAs (si-Has2os-#1, si-Has2os-#2, si-Has2os-#3) significantly increased the activity of JNK signaling (Figure 4G), suggesting that Has2os may regulate the differentiation of C2C12 cells by inhibiting JNK signaling activity.

**Figure 5:**
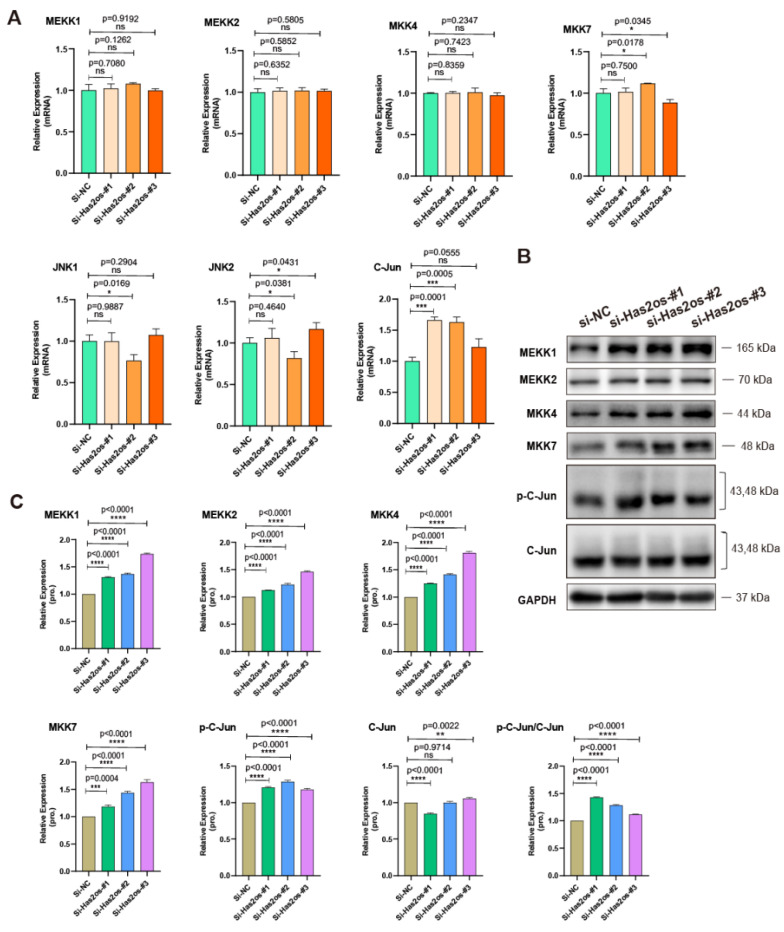
Knockdown of Has2os affected the expression of JNK upstream and downstream factors. Cells Transfected with siRNAs were maintained in growth medium for two days and shifted into DM for another three days, then cells were harvested for analysis. (**A**). qRT-PCR assays of the key factors of the JNK/MAPK signaling (MEKK1, MEKK2, MKK4, MKK7, JNK1, JNK2, and C-JUN) in C2C12 cells that were transfected with si-Has2os or si-NC. The results are normalized to those of with si-NC. (**B**). Western blot analysis shows the protein expression of key factors of JNK/MAPK signaling (MEKK1, MEKK2, MKK4, MKK7, p-C-Jun, and C-Jun). (**C**). Relative expression in (**B**) were calculated. GAPDH was the internal control. The results are normalized to those of with si-NC. Values were presented as means ± SEM. The statistical significance was calculated by *t*-test.

Then, we tested the mRNA levels of the signaling molecules upstream of the JNK/MAPK signaling pathway and found that the mRNA levels of MEKK1, MEKK2, MKK4, and MKK7 did not change after si-Has2os. At the same time, there was no significant change in the mRNA expression levels of JNK1 and JNK2. However, the mRNA of the signaling molecule C-Jun downstream of the JNK/MAPK signaling pathway was significantly elevated (Figure 5A). At the same time, the protein levels of MEKK1, MEKK2, MKK4, MKK7, p-C-Jun, and C-Jun after si-Has2os were determined by Western blot, and the protein levels of MEKK1, MEKK2, MKK4, MKK7, and p-C-Jun were significantly upregulated (*p* < 0.0001, Figure 5B). Quantitative analysis of protein conduction also showed a significant increase in protein levels in MEKK1, MEKK2, MKK4, and MKK7 (*p* < 0.0001, Figure 5C). Using the measured protein gray value, the ratio of p-C-Jun to C-Jun was obtained, and it was found that the ratio after si-Has2os was higher than that of the si-NC group (*p* < 0.0001, Figure 5C). The above results validated that Has2os negatively regulates the JNK/MAPK signaling pathway.

### 3.5. The lncRNA Has2os Is Involved in the Early Repair of the Muscle Injury

**Figure 6:**
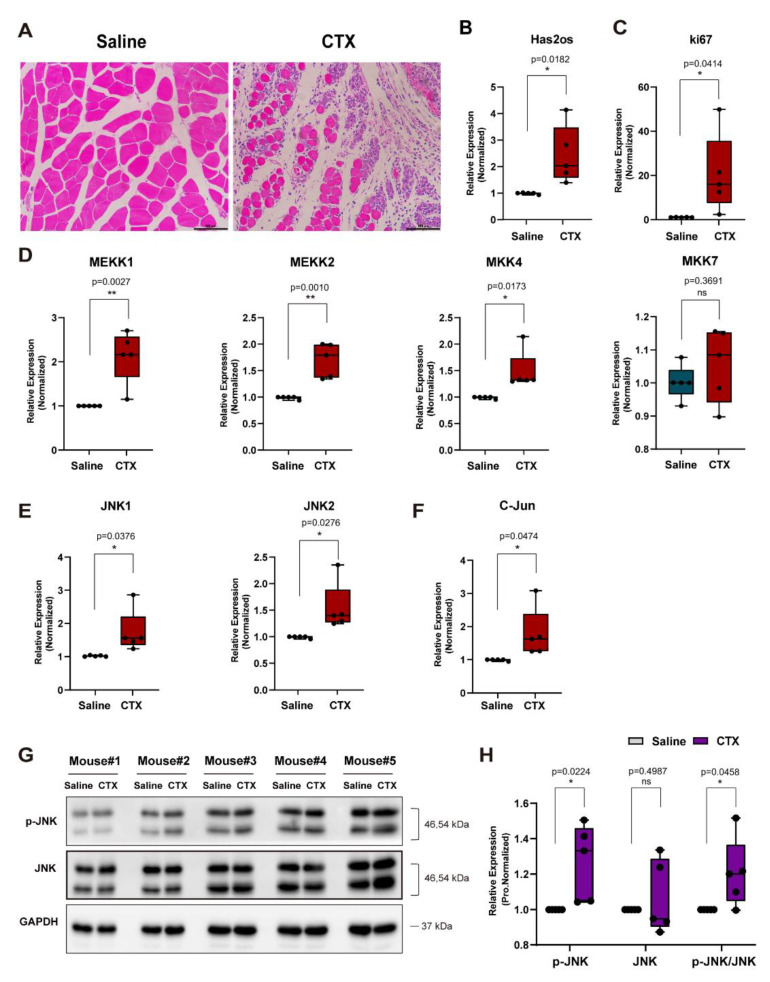
Has2os was involved in the early repair of muscle injury. (**A**). Immunohistochemistry results showed the morphology of injured muscle induced by CTX for 3 days. Scale bar = 100 μm. (**B**). The expression levels of Has2os after muscle injury. (**C**) The mRNA expression levels of ki67 after muscle injury. (**D–F**). qRT-PCR assays of the key factors of the JNK/MAPK signaling (MEKK1, MEKK2, MKK4, MKK7, JNK1, JNK2, and c-JUN) after muscle injury. (**G**). Western blot analysis shows the protein expression of p-JNK and JNK after muscle injury. (**H**). Relative expression in (**G**) were calculated. GAPDH was the internal control. The results are normalized to those of with si-NC. Values were presented as means ± SEM. The statistical significance was calculated by *t*-test.

Furthermore, we contemplated whether Has2os was involved in skeletal muscle regeneration. Firstly, we constructed a mice muscle regeneration model by CTX (Cardiotoxin), separately injecting five mice. The immunohistochemistry results, as shown in Figure 6A, demonstrate that muscle tissue injected CTX with large numbers of accumulating inflammatory cells, and tissue cells were in the early stage of repair. The RNA expression levels of Has2os and ki67 after muscle injury were then measured by qRT-PCR. As shown in Figure 6B,C, the results demonstrate that the RNA expression levels of Has2os and ki67 are higher than those of the control group, which not only proves that muscle satellite cells are activated and proliferated in the early muscle tissue of muscle injury repair, but also that Has2os is involved in early repair after muscle injury. To explore the changes in the JNK/MAPK signaling pathway during impaired muscle regeneration, we first examined the mRNA levels of the upstream signaling molecules of the JNK/MAPK signaling pathway and found that the mRNA levels of MEKK1, MEKK2, MKK4, and MKK7 were significantly upregulated (Figure 6D). In addition, the results show that the mRNA levels of JNK1, JNK2, and its downstream factor C-Jun were significantly increased (Figure 6E,F). Then, we used Western blot to analyze the p-JNK and JNK proteins expression in these mouse tissue and found that the p-JNK protein levels were significantly upregulated, whereas JNK protein levels only changed slightly (Figure 6G). The protein gray calculated value also showed that the ratios of p-JNK to JNK were greater than those of the control saline group (Figure 6H). The above results suggest that both Has2os and the JNK/MAPK kinases cascade are involved in the early repair of muscle damage.

## 4. Discussion

Increasing evidence suggests that lncRNAs represent a new class of regulators of skeletal muscle homeostasis [13,18]. In this study, we uncovered a new function of lncRNA Has2os, which is highly expressed in skeletal muscle and upregulated during myogenesis, and knockdown of Has2os dramatically blocks myogenesis and promotes proliferation. We further demonstrated that lncRNAs Has2os regulates myogenesis and regeneration via the JNK/MAPK signaling pathway. Our studies provided the first evidence that lncRNA and the Has2os-JNK/MAPK axis both underwent highly dynamic changes during muscle differentiation and regeneration, which highlights a potential linkage between lncRNAs and the signaling pathway that functions in this physiology process.

Notably, in this study, we demonstrate that knocked-down Has2os blocked myogenesis and promoted proliferation by activating JNK/MAPK signaling, accompanied with the upregulated protein expression of p-JNK, its upstream factors (MEKK1, MEKK2, MKK4, and MKK7), and its downstream factor, p-C-Jun. This finding is consistent with our previous report that JNK/MAPK signaling was downregulated during skeletal muscle development in vivo and in vitro, and JNK/MAPK signaling pathway inhibited muscle differentiation [7]. In fact, the JNK/MAPK kinases cascades are too complicated to elucidate, and there are some limitations to our study. Similar to the mRNA and protein analysis in the Has2os-depleted cells, the mRNA expression levels of genes involved in JNK cascades almost did not change, but the protein levels were significantly elevated (Figure 5A–C), indicating that Has2os negatively regulates the JNK/MAPK signaling pathway by inhibiting the phosphorylation of JNK in a post-transcription manner. However, when we tested mRNAs and protein expression in the CTX injury mice, not only is the p-JNK protein increased, but so are all mRNAs of the JNK upstream and downstream factors (Figure 6D–H). On the one hand, this might be due to the complexity of in vivo models. In the present study, we examined the early stage of regeneration to obtain the injury TA muscle on the third day post-CTX treatment. At this time point, MuSC (muscle stem cells) were actively proliferating and starting differentiation, meaning that both extreme proliferation and differentiation extreme occur [35], accompanied by various signaling pathway changes, including JNK/MAPK signaling. On the other hand, it is worth noting that expression of some genes could be induced early after transfection but reduced quickly after that, so it is possible that we missed the RNA expression change time point of some genes in the siRNA samples. Further research is still needed to answer whether the induction of the Has2os results from activation of the JNK/MAPK pathway by the CTX treatment, and whether Has2os-JNK/MAPK axis could form a feedback loop. Greater research efforts regarding the regulation of signaling pathways in this process are critical for regenerative medicine.

One of the interesting findings in this study is that the expression pattern and function of lncRNA Has2os were consistent with its host protein-coding gene Has2. Has2 is highly expressed during muscle differentiation and required for myogenic development and limb formation [29,30]. Additionally, it is reported that Has2 expression is connected with an intracellular signaling cascade consisting of p38, MSK1, c-Fos, and AP-1 [36], which indicates that Has2 expression is regulated by MAPK signaling. Due to the complex characteristics of lncRNAs, it is wise to explore the function and regulatory mechanisms of lncRNAs via their neighboring genes under the hypothesis that lncRNA could regulate the expression of neighboring genes by *cis*-acting mechanisms [33]. Given this robust hypothesis and Has2os/Has2 location, as well as their similar expression pattern and function in skeletal muscle cells, we strongly speculate that there is a connection between lncRNA Has2os and Has2 in the muscle system, which it is worth further study. For example, LncMyoD was identified as a muscle-specific lncRNA that is encoded next to the MyoD gene, directly activated by MyoD during myoblast differentiation, and uncovered the MyoD-LncMyoD-IMP2 axis as a mechanism to elucidate how MyoD blocks proliferation to create a permissive state for differentiation [20]. Subsequently, it is reported that LncMyoD exclusively binds with MyoD and not with other myogenic regulatory factors, promoting the transactivation of target genes [21]. These reports established a double-positive feedback regulatory network between LncMyoD and MyoD. Another MyoD-localized lncRNA linc-MD1 is reported to be expressed during myoblast differentiation and regulate myogenesis by working as a microRNA (miRNA) sponge to govern the time of muscle differentiation [19]. Moreover, lncRNA Myoparr is a myogenin promoter-associated lncRNA, confirmed to be essential both for the specification of myoblasts by activating neighboring myogenin expression and for myoblast cell cycle withdrawal by activating myogenic miRNA expression [22]. Since Has2 are reported to be involved in regulating muscle cell proliferation and limb organ development, it is of great interest to identify and analyze in future research.

Disturbed myogenesis may be a component of the mechanisms of diseases, such as cachexia, sarcopenia, and especially muscular dystrophy. Normal skeletal muscle cells have a robust regenerative response that can generate new muscle fibers, and the skeletal muscle regenerative response in muscular dystrophy patients fails to meet the needs of the repair process after muscle cell degeneration. Existing research results show that lncRNA functions to promote the proliferation, differentiation, and regeneration of skeletal muscle, which can slow down or save muscle atrophy [37,38]. For example, Li, Zhenhui et al. constructed an lncRNA–miRNA–gene network associated with myogenesis and determined that IRS1 can regulate the proliferation and differentiation of myoblasts in vitro, muscle mass, and average muscle fibers in vivo, and regulate the expression of atrophy-related genes to rescue muscle atrophy [39]. This study demonstrated that Has2os has similar functions to IRS1, which not only promotes skeletal muscle differentiation but also participates in repair and regeneration after skeletal muscle injury. The newly identified Has2os-JNK pathway, particularly the tissue-specific Has2os, will provide a potential functional mechanism in contexts of disease and pave the way for future applications in medicine.

## Figures and Tables

**Table 1:** qPCR primers.

Gene Name	Primer	Sequence (5′-->3′)
MyHC	MyHC-RT-qF	CGCAAGAATGTTCTCAGGCT
	MyHC-RT-qR	GCCAGGTTGACATTGGATTG
Mef2C	Mef2C-RT-qF	ATCCCGATGCAGACGATTCAG
	Mef2C-RT-qR	AACAGCACACAATCTTTGCCT
MyoD	MyoD-RT-qF	CTACCCAAGGTGGAGATCCTG
	MyoD-RT-qR	CACTGTAGTAGGCGGTGTCGT
MyoG	MyoG-RT-qF	ATCCAGTACATTGAGCGCCTAC
	MyoG-RT-qR	GACGTAAGGGAGTGCAGATTGT
Has2os	Has2os-RT-qF	CTCCGGAGCCCCTTTGAC
	Has2os-RT-qR	ACGCGTTTGGGTGAACTTTG
Has2	Has2-RT-qF	CATCTGTGGAGATGGTGAAGGTC
	Has2-RT-qR	AGCCATCCAGTATCTCACGCTG
ki67	ki67-RT-qF	GAGGAGAAACGCCAACCAAGAG
	ki67-RT-qR	TTTGTCCTCGGTGGCGTTATCC
Pax7	Pax7-RT-qF	GTTCGGGAAGAAAGAGGACGAC
	Pax7-RT-qR	GGTTCTGATTCCACATCTGAGCC
JNK1	JNK1-RT-qF	CGTTCTCCGCTACGGGCTT
	JNK1-RT-qR	AGTCTAATGGCTTCATCCAAATA
JNK2	JNK2-RT-qF	CTGGAGCCCAAGGAATTGT
	JNK2-RT-qR	GCGTTTGGTTCTGAAAAGGA
MEKK1	MEKK1-RT-qF	TCCAGAAGTTTGTGTCACGC
	MEKK1-RT-qR	GGGACACATCTGCTCCTCTT
MEKK2	MEKK2-RT-qF	CCCAGAGTATGACGACAGTCGA
	MEKK2-RT-qR	GGTAGACCCTACCAAAAGCTCC
MKK4	MKK4-RT-qF	TAAACCGTGCTGTCGATTTG
	MKK4-RT-qR	TCGGACCTCCAGCTTCTG
MKK7	MKK7-RT-qF	GAAGCAGGAGAACCGTGAGG
	MKK7-RT-qR	AGGAGCAGGGCTTAGAGTG
C-Jun	C-Jun-RT-qF	GAAAAGTAGCCCCCAACCTC
	C-Jun-RT-qR	GGGACACAGCTTTCACCCTA
GAPDH	GAPDH-RT-qF	CGTCCCGTAGACAAAATGGT
	GAPDH-RT-qR	TCAATGAAGGGGTCGTTGAT

**Table 2:** Sequences of siRNAs.

Gene Name	Sense (5′-->3′)	Antisense (5′-->3′)
si-NC	UUCUCCGAACGUGUCACGUTT	ACGUGACACGUUCGGAGAATT
si-Has2os-#1	GGAGUAUUGAUGCAGGCAATT	UUGCCUGCAUCAAUACUCCTT
si-Has2os-#2	GGGACAAUCAGCUUUCCUUTT	AAGGAAAGCUGAUUGUCCCTT
si-Has2os-#3	GCGUCUGUAUAACGUACCUTT	AGGUACGUUAUACAGACGCTT

## Data Availability

All data are available in the main text or the Supplementary Materials.

## Supplementary Materials

The following supporting information can be downloaded at https://www.mdpi.com/article/10.3390/cells11213497/s1 File S1: Has2os_MAF_file; Figure S1: Has2 expression increased in differentiated muscle cells; Figure S2: Has2 expression levels in myoblasts were not altered when Has2os was knocked down; Figure S3: Both the mRNA and protein expression levels of Pax7 and ki67 were promoted after Has2os knockdown.

## References

[1] Christ B., Brand-Saberi B. (2002). Limb muscle development. Int. J. Dev. Biol..

[2] Cong X.X., Gao X.K., Rao X.S., Wen J., Liu X.C., Shi Y.P., He M.Y., Shen W.L., Shen Y., Ouyang H. (2020). Rab5a activates IRS1 to coordinate IGF-AKT-mTOR signaling and myoblast differentiation during muscle regeneration. Cell Death Differ..

[3] Bentzinger C.F., Wang Y.X., Rudnicki M.A. (2012). Building Muscle: Molecular Regulation of Myogenesis. Cold Spring Harb. Perspect. Biol..

[4] Bryson-Richardson R.J., Currie P. (2008). The genetics of vertebrate myogenesis. Nat. Rev. Genet..

[5] Moncaut N., Rigby P.W.J., Carvajal J.J. (2013). Dial M(RF) for myogenesis. FEBS J..

[6] Le Grand F., Rudnicki M.A. (2007). Skeletal muscle satellite cells and adult myogenesis. Curr. Opin. Cell Biol..

[7] Xie S.-J., Li J.-H., Chen H.-F., Tan Y.-Y., Liu S.-R., Zhang Y., Xu H., Yang J.-H., Liu S., Zheng L.-L. (2018). Inhibition of the JNK/MAPK signaling pathway by myogenesis-associated miRNAs is required for skeletal muscle development. Cell Death Differ..

[8] Liu S., Xie S., Chen H., Li B., Chen Z., Tan Y., Yang J., Zheng L., Xiao Z., Zhang Q. (2021). The functional analysis of transiently upregulated miR-101 suggests a “braking” regulatory mechanism during myogenesis. Sci. China Life Sci..

[9] Xie S.J., Lei H., Yang B., Diao L.T., Liao J.Y., He J.H., Xiao Z.D. (2021). Dynamic m(6)A mRNA Methylation Reveals the Role of METTL3/14-m(6)A-MNK2-ERK Signaling Axis in Skeletal Muscle Differentiation and Regeneration. Front Cell Dev. Biol..

[10] Diao L.-T., Xie S.-J., Yu P.-J., Sun Y.-J., Yang F., Tan Y.-Y., Tao S., Hou Y.-R., Zheng L.-L., Xiao Z.-D. (2021). N6-methyladenine demethylase ALKBH1 inhibits the differentiation of skeletal muscle. Exp. Cell Res..

[11] Chen L., Carmichael G.G. (2010). Long noncoding RNAs in mammalian cells: What, where, and why?. Wiley Interdiscip. Rev. RNA.

[12] Wang K.C., Chang H.Y. (2011). Molecular Mechanisms of Long Noncoding RNAs. Mol. Cell.

[13] Wang S., Jin J., Xu Z., Zuo B. (2019). Functions and Regulatory Mechanisms of lncRNAs in Skeletal Myogenesis, Muscle Disease and Meat Production. Cells.

[14] Li Y., Chen X., Sun H., Wang H. (2018). Long non-coding RNAs in the regulation of skeletal myogenesis and muscle diseases. Cancer Lett..

[15] Milligan L., Antoine E., Bisbal C., Weber M., Brunel C., Forné T., Cathala G. (2000). H19 gene expression is up-regulated exclusively by stabilization of the RNA during muscle cell diferentiation. Oncogene.

[16] Cai B., Ma M., Zhang J., Kong S., Zhou Z., Li Z., Abdalla B.A., Xu H., Zhang X., Lawal R.A. (2022). Long noncoding RNA ZFP36L2-AS functions as a metabolic modulator to regulate muscle development. Cell Death Dis..

[17] Lv W., Jin J., Xu Z., Luo H., Guo Y., Wang X., Wang S., Zhang J., Zuo H., Bai W. (2020). lncMGPF is a novel positive regulator of muscle growth and regeneration. J. Cachex-Sarcopenia Muscle.

[18] Martone J., Mariani D., Desideri F., Ballarino M. (2020). Non-coding RNAs Shaping Muscle. Front. Cell Dev. Biol..

[19] Cesana M., Cacchiarelli D., Legnini I., Santini T., Sthandier O., Chinappi M., Tramontano A., Bozzoni I. (2011). A long noncoding RNA controls muscle differentiation by functioning as a competing endogenous RNA. Cell.

[20] Gong C., Li Z., Ramanujan K., Clay I., Zhang Y., Lemire-Brachat S., Glass D.J. (2015). A Long Non-coding RNA, LncMyoD, Regulates Skeletal Muscle Differentiation by Blocking IMP2-Mediated mRNA Translation. Dev. Cell.

[21] Dong A., Preusch C.B., So W.K., Lin K., Luan S., Yi R., Cheung T.H. (2020). A long noncoding RNA, LncMyoD, modulates chromatin accessibility to regulate muscle stem cell myo-genic lineage progression. Proc. Natl. Acad. Sci. USA.

[22] Hitachi K., Nakatani M., Takasaki A., Ouchi Y., Uezumi A., Ageta H., Inagaki H., Kurahashi H., Tsuchida K. (2019). Myogenin promoter-associated lncRNA Myoparr is essential for myogenic differentiation. EMBO Rep..

[23] Sui Y., Han Y., Zhao X., Li D., Li G. (2019). Long non-coding RNA Irm enhances myogenic differentiation by interacting with MEF2D. Cell Death Dis..

[24] Lu L., Sun K., Chen X., Zhao Y., Wang L., Zhou L., Sun H., Wang H. (2013). Genome-wide survey by ChIP-seq reveals YY1 regulation of lincRNAs in skeletal myogenesis. EMBO J..

[25] Zhou L., Sun K., Zhao Y., Zhang S., Wang X., Li Y., Lu L., Chen X., Chen F., Bao X. (2015). Linc-YY1 promotes myogenic differentiation and muscle regeneration through an interaction with the transcription factor YY1. Nat. Commun..

[26] Wang L., Zhao Y., Bao X., Zhu X., Kwok Y.K.-Y., Sun K., Chen X., Huang Y., Jauch R., Esteban M.A. (2015). LncRNA Dum interacts with Dnmts to regulate Dppa2 expression during myogenic differentiation and muscle regeneration. Cell Res..

[27] Yu X., Zhang Y., Li T., Ma Z., Jia H., Chen Q., Zhao Y., Zhai L., Zhong R., Li C. (2017). Long non-coding RNA Linc-RAM enhances myogenic differentiation by interacting with MyoD. Nat. Commun..

[28] Xie S.-J., Tao S., Diao L.-T., Li P.-L., Chen W.-C., Zhou Z.-G., Hu Y.-X., Hou Y.-R., Lei H., Xu W.-Y. (2021). Characterization of Long Non-coding RNAs Modified by m6A RNA Methylation in Skeletal Myogenesis. Front. Cell Dev. Biol..

[29] Matsumoto K., Li Y., Jakuba C., Sugiyama Y., Sayo T., Okuno M., Dealy C.N., Toole B.P., Takeda J., Yamaguchi Y. (2009). Conditional inactivation of Has2 reveals a crucial role for hyaluronan in skeletal growth, pattern-ing, chondrocyte maturation and joint formation in the developing limb. Development.

[30] Hunt L.C., Gorman C., Kintakas C., McCulloch D.R., Mackie E.J., White J.D. (2013). Hyaluronan synthesis and myogenesis: A requirement for hyaluronan synthesis during myogenic dif-ferentiation independent of pericellular matrix formation. J. Biol. Chem..

[31] Gesteira T.F., Sun M., Coulson-Thomas Y.M., Yamaguchi Y., Yeh L.K., Hascall V., Coulson-Thomas V.J. (2017). Hyaluronan Rich Microenvironment in the Limbal Stem Cell Niche Regulates Limbal Stem Cell Dif-ferentiation. Investig. Ophthalmol. Vis. Sci..

[32] Simpson R.M., Hong X., Wong M.M., Karamariti E., Bhaloo S.I., Warren D., Kong W., Hu Y., Xu Q. (2016). Hyaluronan Is Crucial for Stem Cell Differentiation into Smooth Muscle Lineage. Stem Cells.

[33] Lee J.T. (2012). Epigenetic Regulation by Long Noncoding RNAs. Science.

[34] Burattini S., Ferri P., Battistelli M., Curci R., Luchetti F., Falcieri E. (2004). C2C12 murine myoblasts as a model of skeletal muscle development: Morpho-functional characterization. Eur. J. Histochem..

[35] Shang M., Cappellesso F., Amorim R., Serneels J., Virga F., Eelen G., Carobbio S., Rincon M.Y., Maechler P., De Bock K. (2020). Macrophage-derived glutamine boosts satellite cells and muscle regeneration. Nature.

[36] Terazawa S., Nakano M., Yamamoto A., Imokawa G. (2020). Mycosporine-like amino acids stimulate hyaluronan secretion by up-regulating hyaluronan synthase 2 via activation of the p38/MSK1/CREB/c-Fos/AP-1 axis. J. Biol. Chem..

[37] Buonaiuto G., Desideri F., Taliani V., Ballarino M. (2021). Muscle Regeneration and RNA: New Perspectives for Ancient Molecules. Cells.

[38] Huang H., Xing D., Zhang Q., Li H., Lin J., He Z., Lin J. (2021). LncRNAs as a new regulator of chronic musculoskeletal disorder. Cell Prolif..

[39] Li Z., Cai B., Abdalla B.A., Zhu X., Zheng M., Han P., Nie Q. (2019). LncIRS1 controls muscle atrophy via sponging miR-15 family to activate IGF1-PI3K/AKT pathway. J. Cachexia Sarcopenia Muscle.

