# Supplementary material for: Role of lncRNA Has2os in Skeletal Muscle Differentiation and Regeneration
Source: Cells. 2022 Nov 4;11(21):3497. doi: 10.3390/cells11213497 (PMC9655701; doi:10.3390/cells11213497)
Supplement: Supplementary file 1 [file cells-11-03497-s001.zip › Supplementary Figure-cells-1898682_V1.pdf]

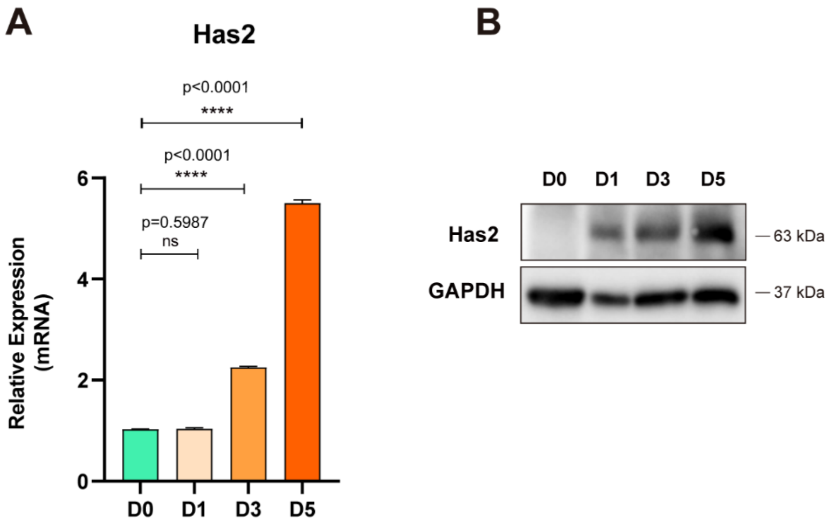

Supplementary Figure S1. Has2 expression increased in diferentiated muscle cells.

(**A**). The mRNA expression levels of Has2 in D0, D1, D3, and D5. D0 represents cells in growth medium and D1, D3, and D5 represent cells switched into differentiation medium for 1, 3, or 5 days. (**B**). The protein expression levels of Has2 were detected by western blotting. GAPDH was the internal control. The statistical significance was calculated by *t*-test.

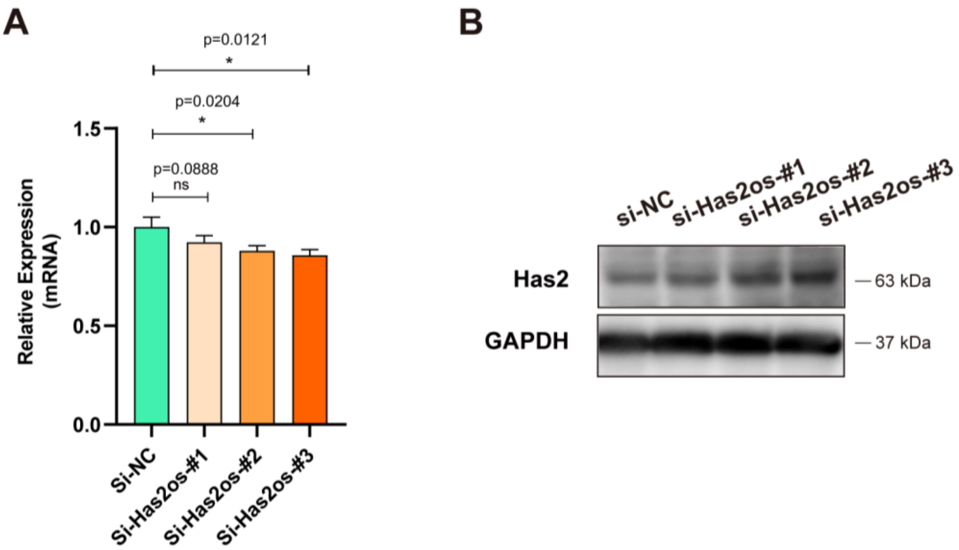

Supplementary Figure S2. Has2 expression levels in myoblasts were not altered when Has2os knockdown

(**A**). The expression levels of Has2 when Has2os knockdown. (**B**). The protein expression levels of Has2 after si-Has2os transfecting were detected by western blot. GAPDH was the internal control. Values are were presented as means ±SEM. The statistical significance was calculated by *t*-test.

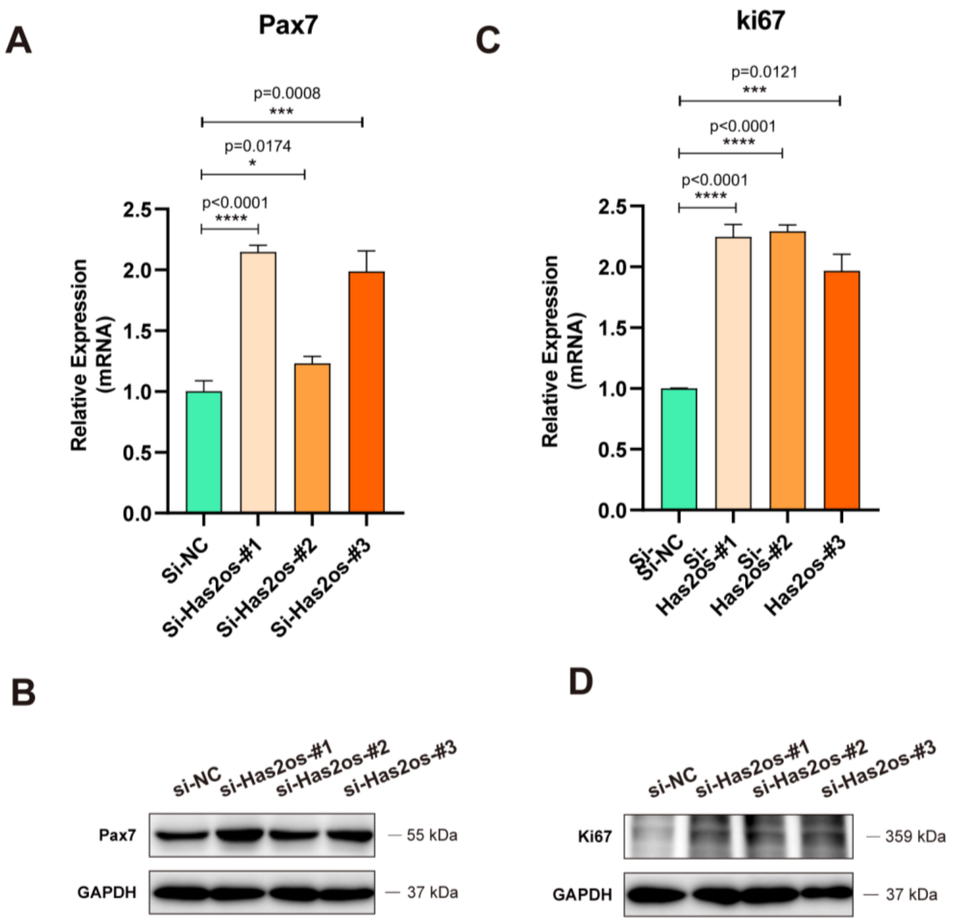

Supplementary Figure S3. Both the mRNA and protein expression levels of pax7 and ki67 were promoted after Has2os knockdown

(**A,** C). The mRNA expression levels of Pax7 and ki67 after Has2os knockdown. (**B, D**). The protein expression levels of Pax7 and ki67 after si-Has2os were detected by western blot. GAPDH was the internal control. Values were presented as means ±SEM. The statistical significance was calculated by *t*-test.
